# Radiologically isolated syndrome in the spectrum of multiple sclerosis

**DOI:** 10.1177/13524585241245306

**Published:** 2024-04-15

**Authors:** Darin T Okuda, Christine Lebrun-Frénay

**Affiliations:** Neuroinnovation Program, Multiple Sclerosis & Neuroimmunology Imaging Program, Department of Neurology, The University of Texas Southwestern Medical Center, Dallas, TX, USA; The University of Texas Southwestern Medical Center, Peter O’Donnell Jr. Brain Institute, Dallas, TX, USA; Université Nice Cote d’Azur, CRCSEP, Nice, France

**Keywords:** Radiologically isolated syndrome, disease-modifying therapy, multiple sclerosis

## Abstract

The radiologically isolated syndrome (RIS) currently represents the earliest detectable preclinical phase of multiple sclerosis (MS). Remarkable advancements have been recently made, including the identification of risk factors for disease evolution, revisions to the existing 2009 RIS criteria, and our understanding of the impact of early disease-modifying therapy use in the prevention/delay of symptomatic MS from two randomized clinical trials. Here, we discuss RIS in the context of the spectrum of MS, implications in the clinical management of individuals, and provide insights into future opportunities and challenges given the anticipated inclusion of asymptomatic MS in the formal definition of MS.

## Introduction

The ancient Greek physician Hippocrates emphasized the importance of observation and clinical findings to understand the basis of disease and facilitate early intervention. Identifying the exact cause and timing of the development of clinical manifestations that follow remain elusive for many disorders, including multiple sclerosis (MS), a heterogeneous autoimmune condition affecting myelin and axons within key anatomical structures within the central nervous system (CNS).

**Figure 1.**
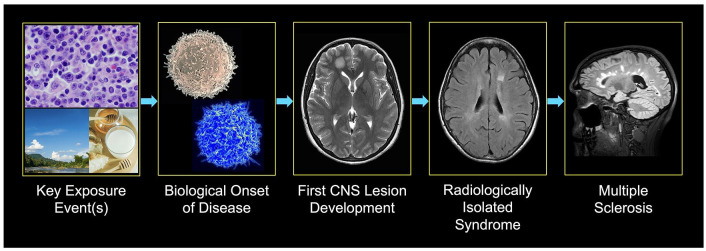
Envisioned series of events from environmental exposures, including the Epstein Barr virus^6^ (top), activation of B and T (blue) immune cells (scanning electron micrographs of human lymphocytes from the National Institute of Allergy and Infectious Diseases), development of the first MRI lesion visible on MRI, and possible evolution to radiologically isolated syndrome and multiple sclerosis.

A prevailing hypothesis for MS involves the activation of the immune system in susceptible individuals by an environmental source, resulting in aberrant responses to normal tissue. Following the biological onset of the disease, resultant microscopic regions of pathological change that remain below the resolution of conventional diagnostic applications may ensue. At the start of the first macroscopic change, a single focus of high-signal abnormality within the brain or spinal cord that is observable on magnetic resonance imaging (MRI) may follow in the absence of symptoms. Whether these two phenomena are temporally independent is not entirely clear. The subsequent development of additional lesions may follow and be identified incidentally on MRI. In such subjects who lack clinical symptoms typical of CNS demyelination and have the requisite number of lesions to fulfill dissemination in space criteria, the classification of radiologically isolated syndrome (RIS) is assigned.^1^ In the era when the syndrome was first named, other independent cohorts were reported that revealed consistent findings of risk for future disease advancement.^2,3^ In collaboration, these groups demonstrated the time course of evolving to clinical MS and associated risk factors.^4,5^ Figure 1 provides a generalized framework for the spectrum of events that may lead to the development of MS.

A clear spectrum of incidental anomalies observed on MRI exists within the CNS. On one hand, observably prominent focal areas of high-signal abnormality coupled with tissue atrophy from autoimmune inflammation are present, and on the other hand, punctate foci resulting from microvascular injury. Given this and real-life situations in which these two processes may be coinciding, continued vigilance in the precise recognition of subjects with white matter anomalies highly suggestive of MS will forever be required. The introduction and application of new imaging techniques have also been of benefit in improving lesion specificity.^7-12^ When accurately recognized with the exclusion of MS mimics, many questions remain, including the classification of subjects based on existing criteria and the best clinical surveillance and treatment practices relying on existing data. Here, we briefly review time-relevant topics in RIS, including the recent change to the original 2009 criteria, the prodromal phase, and the findings from the two recent therapeutic trials in the United States, Europe, and Türkiye, with implications for care. Opportunities for future RIS research in the context of anticipated revisions to the MS criteria will also be discussed.

## RIS

Over the past 5 years, recent advances in our understanding of RIS have occurred. The incidental observation of CNS anomalies has been well-recognized in adults and children.^13^ In addition, risk factors for a first inflammatory demyelinating event, acute or progressive, have been identified, including younger age, gadolinium enhancement, infratentorial/spinal cord lesions, and the presence of cerebrospinal fluid (CSF)–restricted oligoclonal bands within the CNS.^4,5,14^ Recently, insights have been gained on the impact of early treatment in adults, leading to a better understanding of the potential benefits of early treatment in suppressing a first acute clinical event. In addition, recognizing individuals who failed to meet the 2009 RIS criteria^1^ based on the more stringent MRI spatial requirements in comparison to the current McDonald^15^ standard was a meaningful observation in the study of RIS during this period, enabling the recognition and examination of an earlier group that may be vulnerable to disease advancement not accounted for by the existing criteria. The study of the natural history of such individuals resulted in revisions to the original 2009 RIS criteria in 2023, incorporating accessible clinical measures that could be implemented in diverse healthcare settings.^16^

What do we know about those individuals who almost fulfill the 2009 RIS criteria that are now included in the 2023 criteria? In the study aimed at updating the criteria for RIS of 747 individuals with incidentally identified anomalies within the CNS, 251 (33.6%) did not meet 2009 RIS criteria for dissemination in space, having fewer lesions than required.^16^ Significant differences between those meeting 2023 RIS criteria were observed in that they (1) were identified as being younger, (2) were more likely to develop new T2-weighted hyperintense lesions over time, and (3) at 5 years from the first abnormal MRI scan had a lower cumulative probability of 29.0% for a first clinical event when compared to those meeting 2009 RIS criteria (38.7%). The rate of evolution to a first neurological event for those falling short of the 2009 RIS criteria increased to 38% if the setting of observed spinal cord lesions or CSF-restricted oligoclonal bands were present. Based on observations from this work, 2023 RIS criteria (sensitivity: 86.0%, specificity: 35.4%) were proposed that encompassed those fulfilling the original 2009 criteria in addition to individuals having at least one juxtacortical, periventricular, infratentorial, or spinal cord lesion and meeting two of three additional criteria: (1) presence of abnormal CSF-restricted oligoclonal bands, (2) presence of at least one spinal cord lesion consistent with inflammatory demyelination, and/or (3) evidence of dissemination in time on any follow-up MRI defined by the presence of one or more new T2-weighted hyperintensities or gadolinium enhancement typical for MS (within the brain given the original incidental anomaly was a spinal cord lesion). Table 1 provides a summary of the 2009 and 2023 RIS criteria.

**Table 1.** Summary of the 2009 and 2023 RIS criteria.

2009 RIS criteria^a^	2023 RIS criteria^a^
Incidentally identified CNS white matter lesions that appear typical for inflammatory demyelination with at least three of the following required to fulfill dissemination in space criteria: • >9 T2-weighted hyperintense lesions or ⩾1 gadolinium-enhancing lesion • ⩾1 juxtacortical lesion • ⩾1 infratentorial lesion • ⩾3 periventricular lesions	Fulfillment of 2009 RIS dissemination in space criteria OR The presence of at least one lesion in a location^b^ typical for multiple sclerosis and two of the three following factors: • Spinal cord lesion • Cerebrospinal fluid–restricted oligoclonal bands • New asymptomatic T2 or gadolinium-enhancing lesion demonstrating dissemination in time

RIS: radiologically isolated syndrome; CNS: central nervous system.

a No historical reports of remitting clinical symptoms consistent with neurological dysfunction and observed MRI anomalies are not better accounted for by another disease process.

b Juxtacortical, periventricular, infratentorial, or spinal cord.

The revised criteria were introduced to establish a more broadly applicable framework for clinicians, aligning with the existing 2017 McDonald Criteria while enhancing sensitivity and maintaining specificity.^15^ With this revision, an increase in the number of individuals recognized is anticipated, but the overall magnitude of included individuals over prior criteria remains unknown. This study also intended to generate data that would be of value in the counseling and medical management of individuals with incidental anomalies earlier in the phase of the disease. Despite the absence of established guidelines for medical work-up, clinical follow-up, and MRI surveillance, recent proposed guidance involved obtaining baseline cervical and thoracic imaging in the presence of an abnormal brain MRI study along with annual clinic and imaging surveillance.^17^ Continued exploration for alternate explanations for the incidentally identified abnormal MRI findings should also persist.

## Prodromal phase of MS and RIS

A higher utilization rate of healthcare resources has been described in people prior to the diagnosis of MS, suggesting the existence of a prodromal phase or the presence of non-specific symptom development that precedes classical experiences (e.g. vision loss, motor weakness, paresthesias, etc.) by people with MS.

The prospect that non-specific symptoms may represent an earlier indicator for the diagnosis of MS is compelling. If true, individuals who would benefit from rigorous surveillance and preventive treatment could be identified even earlier in the MS disease spectrum. In earlier work, 98% of those who were eventually diagnosed with MS averaged at least one physician visit per year in comparison to 87% of individuals within a matched population.^18^ Annual healthcare use was also observed to increase 5 years and 1 year before the first demyelinating disease claim.^19^ A higher rate of psychiatric comorbidity has also been reported within the prodromal period, along with symptoms of fatigue, sleep disorders, anemia in men, and pain.^20,21^

More refined data are needed to understand these findings better. First, MRI data were not provided on the group that later developed MS, creating uncertainty as to whether structural lesions characteristic of MS or non-specific white matter disease were already present. Neurologists frequently consult on individuals with abnormal brain or spinal cord MRI studies related to non-specific white matter disease. Such individuals have also described an abundance of non-neurological and neurological symptoms. Elevated serum levels of neurofilament light chain were also found among military personnel who later developed MS.^22^ Although this biomarker is associated with other neurodegenerative diseases, the observation of elevated levels may identify a subgroup of individuals who may benefit from neuroimaging exploration. Second, the identified people with MS were acquired through healthcare administrative data codes. Accompanying clinical records were also not verified for the diagnosis of MS. Third, as MS is an autoimmune condition, the coexistence or overlap of other autoimmune disorders during the period leading up to the first diagnostic code is highly plausible, resulting in increased healthcare resource utilization over time. Such experiences may not represent prodromal experiences but concomitant autoimmune phenomena.

In the original study describing those with RIS,^1^ individuals were imaged for a variety of reasons, with headache and trauma being the most common reasons for an initial MRI scan. Other reasons for imaging included family history for MS, galactorrhea, aneurysm screen for polycystic kidney disease, dysmenorrhea/amenorrhea, and research control data. Other reasons included anxiety/panic attacks, abdominal/back pain, and hypersomnolence, reasons that appear to be more aligned with what has been described within the MS prodrome. However, non-specific symptoms that define the prodromal phase of MS may represent specific symptoms related to the disease. In the case of generalized pain or headache, a spinal cord lesion may impact emanating nerves, resulting in muscle tightness at the back or, if a high cervical lesion is present, occipital discomfort that may result in head pain. Mood alteration (i.e. dysphoria, anxiety, irritability, etc.) and the need for psychiatric care based on these symptoms may represent an organic symptom of MS. The findings on MRI with frontotemporal involvement in MS have been associated with euphoria^23^ and hippocampal^24^ involvement with depression, highlighting that these symptoms, although generalized, may have greater specificity for MS when distinct areas of the CNS are affected.

## Therapeutic trials in RIS

The first therapeutic trial in RIS, ARISE, involved treatment with dimethyl fumarate (Tecfidera^®^) to evaluate the efficacy and safety of this US Food and Drug Administration–approved therapy in people with RIS. The primary endpoint of the study was the onset of the first clinical symptom attributable to a CNS demyelinating event over 96 weeks. Eighty-seven individuals were recruited across multiple centers within the United States and randomized 1:1 to the active treatment arm or placebo using the same treatment regimens utilized in the original pivotal trials.^25,26^ Following treatment with dimethyl fumarate, the risk of a first clinical demyelinating event during the 96 weeks was significantly reduced by 82% in the unadjusted and 93% in the adjusted models. The results from a second randomized controlled trial, TERIS, involving teriflunomide (Aubagio^®^), conducted in Europe and Türkiye, quickly followed, having the same primary outcome measure as ARISE.^27^ Following the randomization of 89 people with RIS 1:1 to placebo or teriflunomide, treatment benefit was demonstrated with 63% and 72% reduced risk estimates for a first clinical event in the unadjusted and adjusted statistical models. Unexpected safety outcomes were not observed in either trial. These studies showed the benefit of early treatment intervention in the MS disease spectrum.

These trials provided insights related to three key points. First, recruited individuals with RIS were at risk for disease advancement, further supporting the existence of an asymptomatic phase of MS.^28^ Second, early treatment intervention was beneficial in preventing or delaying the onset of a first inflammatory demyelinating event by two approved disease-modifying therapies with different mechanisms of action. Third, treatment was safe in both studies, and documented adverse events were less frequent than those observed within the pivotal studies in relapsing MS. The therapeutic benefits demonstrated by these seminal trials may be transferable to other immunomodulatory or immunosuppressive treatments commonly used to manage MS, but existing safety and efficacy data beyond that of dimethyl fumarate and teriflunomide are lacking. As treatment earlier in the disease spectrum should lead to a more significant effect on the accumulation of disability^29^ and more substantial suppression of secondary progressive states, the findings from these trials underscore the potential to better impact outcomes related to reducing permanent neurological disability. Learnings from current studies assessing the ideal therapeutic approach, whether early use of high-efficacy treatment is superior to treatment escalation, will further refine approaches to manage those early in the disease course.^30^

The practical clinical implications from these trials may result in an expansion of disease-modifying therapies prescribed by clinicians and advanced practice providers, potentially leading to a further rise in medical waste from premature discontinuations.^31^ However, even before these clinical trial data were available, treated subjects were frequently encountered in many countries.^4^ The basis for use is not entirely clear and may have been biased by the findings on MRI. Intuitively, using treatments even with marginal benefit in reducing disease evolution would be of value to an individual with RIS. However, costs to healthcare systems, the risks of adverse reactions from treatment, and imposed life/family planning constraints counterbalanced by the magnitude of clinical benefit are realistic challenges. Not all individuals with RIS will evolve to experience a first neurological symptom.^17,27,32,33^ This is exemplified further by numerous post-mortem investigations revealing signs of incidental histopathological demyelination.^32^ In addition, countervailing factors, including misclassifying MRI lesions as having an origin related to CNS demyelination, are palpable risks.^34^ Despite all best efforts, individuals will be improperly treated.

As MS exacerbations are random events and the time course to a first clinical event may differ from that seen in those with established MS, identifying the best candidates for treatment is essential. Despite not having these data immediately, we can more effectively counsel people with RIS and better understand the clinical and MRI evolution risks. As access to treatment heavily depends on the structure of healthcare systems, how MS is formally defined in the future will heavily influence when and how treatments are used. Unfortunately, even for some, the introduction of treatment even at this early stage of the MS spectrum may be too late in effectively suppressing the onset of a progressive clinical course later in life, given the extent of tissue involvement observed at the time of the index MRI study.^35^

## Future research

Future research in RIS may be significantly impacted by how this clinical course is recognized within the formal criteria for MS. If RIS in specific situations becomes synonymous with MS, access to treatment provided by third-party administrators will be improved for affected people, allowing for more timely access. However, investment by industry in future therapeutic trials that focus on the use of higher efficacy treatments, or those having a specific mechanism of action impacting distinct immune cells within the CNS that better address neurodegeneration, may be substantially reduced given the lack of need for establishing an indication for RIS treatment to healthcare regulators. Many continue to recognize the value in investigating the preceding phases of RIS, given the lack of available treatments to repair CNS injury effectively. Given the contribution of the Epstein–Barr virus to increased risk for the development of MS,^36^ vaccination studies centered around primary disease prevention are to be expected. The search and identification of additional biomarkers that inform future risk for disease advancement is also anticipated.

## Summary

Scientists and clinicians will continue to work toward recognizing the earliest phases of disease for which treatment or clinical surveillance is rational and supported by well-designed studies. Our quest toward (1) identifying those at risk for MS development, (2) refining risk stratification schemes, and (3) intervening at the earliest time frame possible following biological onset requires continuous exploration. Healthcare providers who consult and manage an abundance of people with MS will continue to recognize individuals who may not align precisely with the existing criteria for RIS, even after the release of the recent revisions. The long-term clinical and radiological experience is yet to be defined in these individuals. In total, these efforts move us toward a future that may more rapidly define the disease trajectory for those at risk and those affected by MS.
